# A rare case of giant synovial osteochondromatosis of the thigh: A case report

**DOI:** 10.1097/MD.0000000000018269

**Published:** 2019-12-10

**Authors:** Shuzhong Liu, Xi Zhou, An Song, Zhen Huo, Yipeng Wang, Yong Liu

**Affiliations:** a Department of Orthopedic Surgery, Peking Union Medical College Hospital, Peking Union Medical College and Chinese Academy of Medical Sciences; b Department of Endocrinology, Key Laboratory of Endocrinology, National Health and Family Planning Commission; c Department of Pathology, Peking Union Medical College Hospital, Chinese Academy of Medical Science & Peking Union Medical College, Beijing, China.

**Keywords:** diagnosis, imaging characteristics, surgical treatment, synovial osteochondromatosis, thigh

## Abstract

**Rationale:**

Giant synovial osteochondromatosis of the thigh is a highly unusual disease without standard diagnosis and curative managements so far. Our focus is to report a very rare case of giant synovial osteochondromatosis successfully operated by surgical treatment. The management of these unique cases has certain educational significance in clinical practice.

**Patient concerns:**

A 63-year-old previously healthy man presented to our institution with a 4-year history of continuous progressive hip pain and local numbness of right side in January 2018. One month ago, the patient felt that the above symptoms were aggravated, and the right hip and proximal thigh were significantly swollen.

**Diagnosis:**

Computed tomography and magnetic resonance imaging of the hip revealed the irregular mass in his right thigh. Post-operative pathology confirmed the diagnosis of synovial osteochondromatosis of the thigh.

**Interventions:**

Considering the large volume of the mass and possibility of malignancy, the patient underwent surgical exploration and complete tumor resection.

**Outcomes:**

The patient's neurological deficits and symptoms improved significantly after the surgery, and the postoperative period was uneventful at the 1-year follow-up visit. There were no complications associated with the operation during the follow-up period.

**Lessons:**

Taken together, the lesion's clinical features, imaging results, and pathological characteristics are unique. Synovial osteochondromatosis of the thigh, although rare, should be part of the differential diagnosis when the patient presents with local pain, numbness, swelling or other symptoms. We recommend surgical treatment for the occupying lesion when the tumor has caused symptoms or neurological deficits.

## 1 Introduction

Synovial osteochondromatosis (SOC) is a monoarticular, synovial, proliferative disorder. It is a rare entity which presents with multiple cartilaginous nodules in synovial joints, bursae or tendon sheaths.^[1–3]^ SOC most commonly involves knee joint with a frequency of 50% to 65%.^[1,2]^ Other places that are involved frequently include hip, elbow, shoulder, and ankle joint.^[1–3]^ Observed clinical symptoms were pain, swelling, numbness and limitation of joint movements at the involved area.^[4,5]^ Secondary osteoarthritis findings such as generalized joint effusions, locking, tenderness, and snapping may also occur.^[1–3]^ Therefore, early diagnosis and proper treatment of this unique disease is of great significance. Although rare, SOC should be considered in differential diagnosis of cases presenting with similar symptoms.

To the best of our knowledge, this is a less-documented case of giant synovial osteochondromatosis of the thigh in a man presenting with continuous progressive hip pain, local numbness and swelling, who underwent total resection of the space-occupying lesions. In the follow-up visit, the patient's conditions improved significantly postoperatively. After reviewing pertinent literatures, we discussed common clinical considerations in patients with giant synovial osteochondromatosis of the thigh and management considerations for these patients.

## 2 Case report

A 63-year-old previously healthy man presented to our institution with a 4-year history of continuous progressive hip pain and local numbness of right thigh in January 2018. Upon examining and questioning, the patient stated he has been experiencing a gradual increase in his hip pain, as well as worsening numbness and swelling of the right thigh. In the medical journal of his current illness, the pain in his right hip can reach 4–5 points using the visual analogue scale and cannot be alleviated with rest and hot compresses. One month ago, the patient felt that the above symptoms were aggravated, especially during sleeping and sitting. He denied history of injury and any other underlying diseases. No pertinent family history was identified, including hypertension and cancer.

**Figure 1:**
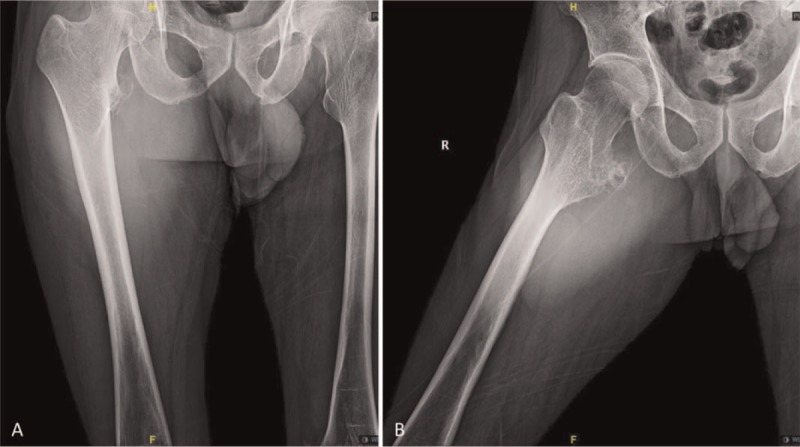
(A, B) Posteroanterior X-ray film of the right hip revealed irregular shadow of a soft tissue mass in the right thigh.

**Figure 2:**
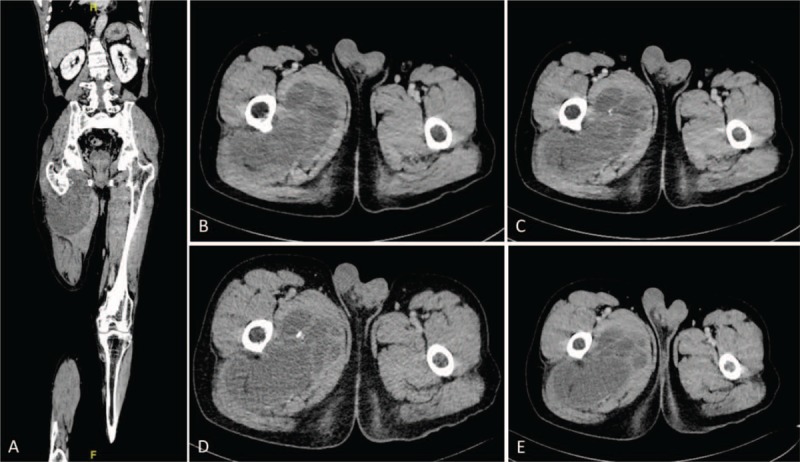
(A–E) Preoperative coronal and transverse CT scan revealed multilocular cystic-solid mass in the right thigh root, with high suspicion of malignancy.

**Figure 3:**
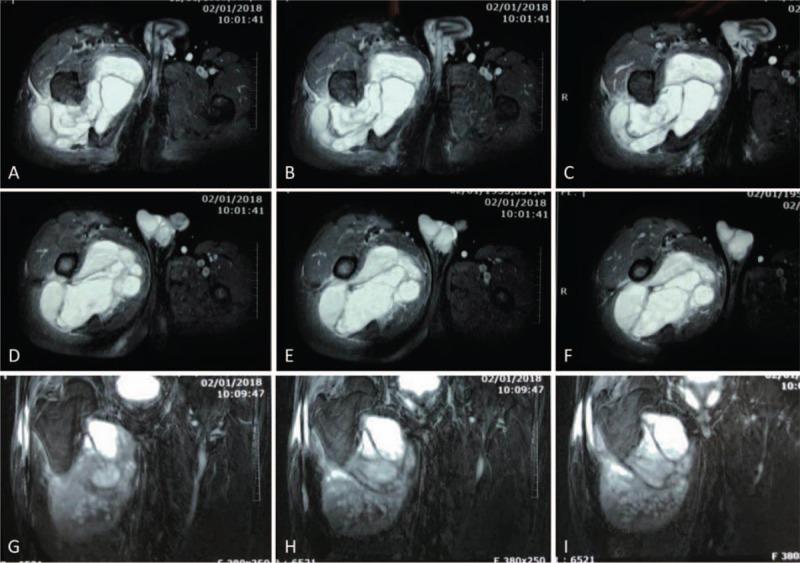
(A–I) Preoperative coronal and transverse MRI scan revealed the irregular mass in his right thigh.

Physical examination showed a mass sized 12 × 24 cm in the proximal thigh, which was hard in texture, unclear in boundary, adherence to surrounding tissues, poor mobility, and no tenderness. However, low skin temperature and varicose vein were not found on the surface of the mass. Routine laboratory studies were almost within normal range, except that the tissue polypeptide specific antigen was significantly elevated to 101.55 U/L (normal: <80 U/L). Plain radiographs showed irregular shadow of a soft tissue mass in his right thigh (Fig. 1A and B). Computed tomography (CT) showed multilocular cystic-solid mass in the right thigh root, with high suspicion of malignancy (Fig. 2A–E). Magnetic resonance imaging (MRI) of the hip revealed the irregular mass in his right thigh mimicking a parosteal sarcoma (Fig. 3A–I).

**Figure 4:**
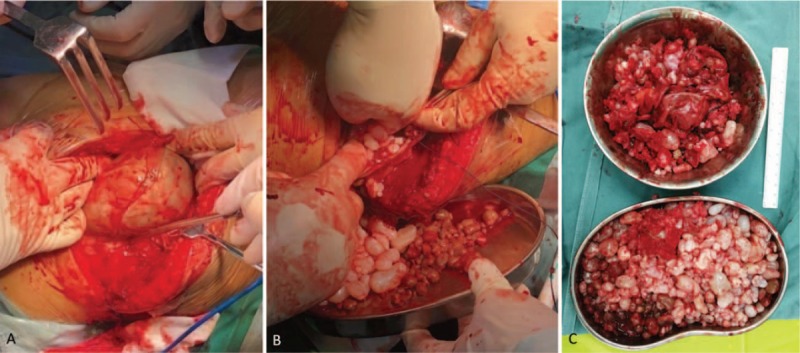
(A, B) Intraoperative photography depicting the exposed tumor. (C) Intraoperative photography depicting totally resected synovial osteochondromatosis.

**Figure 5:**
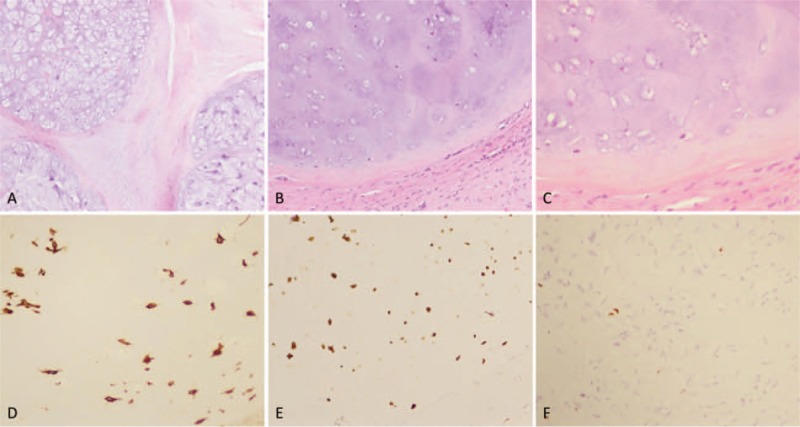
Pathologic histology of tumor specimens. (A–C) Microphotography showing characteristic nests of tumor cells (Zellballen) (H&E, original magnification 40×, 100×, and 200×). (D, E) Immunohistochemistry of the lesion showed Vimentin and S100 positive staining. (F) Ki-67 immunostaining shows 5% Ki-67 positive cells. Ki-67 staining is localized in the tumor nuclei.

Considering the large volume of the mass and possibility of malignancy, surgical exploration and complete tumor resection were performed according to the designed surgical procedure. After successful anesthesia, the patient was placed in a supine position, with the right buttock being cushioned high. During the operation, multiple cystic masses in the deep muscles were seen, with a size of about 24 × 15 × 10 cm extending from the upper middle of the thigh to the right hip (Fig. 4A–C). Each capsule contains clear yellowish and reddish liquids, which are filled with a large number of round, tough, oval-shaped, white translucent, cartilage-like granules with a diameter of about 0.5 to 2.0 cm. The chondroid granules in the capsule were cleaned completely, the wall of the capsule was separated from the surrounding tissues, and the capsule was excised completely and sent for pathological examination. The incision was closed. Intraoperative blood loss was approximately 900 mL, thus we used erythrocyte 2U. The postoperative pathology confirmed the diagnosis of synovial osteochondromatosis of the thigh (Fig. 5A–F). Pathological result was positive for Vimentin and S-100. Biopsy samples were negative for AE1/AE3 and EMA, with 5% Ki-67 positive nuclei.

One week after the operation, the patient's symptoms improved significantly compared to the preoperative status. Postoperatively, visual analogue scale score of his hip pain improved to 0–1 points compared to the preoperative status, 4–5 points. At a 1-year follow-up visit, the patient was doing well, with no local recurrence or new symptoms. There were no complications associated with the operation during the follow-up period.

## 3 Discussion

Synovial osteochondromatosis (SOC) is a benign disorder of nodular cartilaginous neoplastic development of the synovium that can lead to multiple loose bodies within the articular space.^[1–3]^ SOC can be originated from any joint, tendon sheath, or bursae that has synovial tissue, which is characterized by cartilaginous nodule formation secondary to synovial metaplasia.^[1–4]^ Although it is generally progressive in nature, it can limit itself and regress.^[5,6]^ This condition is usually a monoarthritic disease and affects the knee joint in more than 50% of all cases suffering from SOC.^[1–3,7]^ To date, few reports of SOC of the thigh causing clinical symptoms have been documented so far. Therefore, the management of our reported case has certain educational significance in clinical practice. Delay in diagnosis of synovial chondromatosis may occur due to slow progression of disease course and calcification of free cartilage fragments at later stages.^[8,9]^ Clinical manifestations of the patients with SOCs are usually due to mechanical and oppressive effects of the mass. Milgram defined 3 stages of SOC: Stage 1 (early stage) is the active intrasynovial stage during which there is no free articular body. Stage II (mid-term stage) is the transition stage from intrasynovial lesions to free bodies. In this stage, there are both active intrasynovial lesions and free articular bodies. In stage III (late stage), there are multiple free articular bodies in the absence of intrasynovial involvement.^[1–3,10,11]^

The most common radiologic findings, free bodies with varying sizes, can be seen at any place in the tumor cavity.^[12,13]^ Calcification occurs at the last stage and may not be observed in some patients at earlier stage.^[14]^ Intraarticular liquid-like masses, non-calcified masses or swellings which can be differentiated with MRI or CT scan.^[12–15]^ However, difficulties of diagnosis despite CT and MRI should also be considered. In neglected cases or in cases with a long-term disease course, changes in bone or joint cartilage induced by multiple intraarticular lesions, bony erosion, or presence of local osteoporosis make accurate preoperative diagnosis much more difficult.^[16]^ Imaging studies including CT, MRI, bone scan and PET/CT are non-specific, making it difficult to differentiate SOC from other common occupying lesions.^[15–17]^ However, imaging studies may play a crucial role in the decision making of surgical intervention. In our case, the entire size of SOCs was 24 × 20 × 12 cm, and our case had been one of the biggest SOCs ever reported in literature.

Differential diagnosis of synovial osteochondromatosis includes many other occupying diseases. SOC should be differentiated from multiple benign or malignant lesions such as synovial hemangioma, pigmented villonodular synovitis, synovial cyst, osteosarcoma and synovial sarcoma.^[18–21]^ Malignant transformation was reported in few cases at long-term follow-up visit.^[21,22]^ While the literature describes the knee as the most common location of the usual form of SOC, the location in the thigh is extremely a rarity.

The “gold-standard” diagnosis of SOC relies on pathological findings. The main pathological characteristic is chondroid metaplasia of the subintimal tissue of synovial joints.^[1–3,23,24]^ Further reports and analyses of the giant form of SOC are necessary to improve our understanding of this pathological entity and its differences from the usual form to optimize proper clinical management. In our reported case, pathology results showed significant chondroid metaplasia without cellular atypia, which was consistent with synovial osteochondromatosis.

The treatment of first choice for SOC is surgical excision with an open or arthroscopical approach.^[1–3,25–27]^ In the literature, early arthroscopical or open debridement, synoviectomy, and removal of free cartilage masses at an early stage before cartilage damage occurs have shown to be efficient treatments.^[25–27]^ Severity of disease course and affected location should also be considered when surgeons decide whether surgery should be performed.^[25,26]^ Additionally, in some cases that had osteochondroplasty after debridement osteoarthritis did not relapse in 2-years follow up and successful results were achieved.^[25–28]^ Under this circumstance, surgical extent, surgical procedures, and postoperative complications are critical factors that need further investigation. According to our single-center experience, we prefer and recommend open surgery for patients with giant SOCs due to the size of the mass.

In conclusion, this is the first report of giant synovial osteochondromatosis of the thigh in a patient. Although uncommon, synovial osteochondromatosis of the thigh should be part of the differential when the patient presents with atypical symptoms. Surgical treatment is a definite therapy of first choice. Our case highlights the significance of accurate diagnosis and proper treatment for synovial osteochondromatosis. With an accurate diagnosis, proper planning, and accurate surgical manipulation, synovial osteochondromatosis can be diagnosed and managed much more effectively.

## Acknowledgments

We would like to thank our colleagues at the Department of Orthopedic Surgery, Peking Union Medical College Hospital, Chinese Academy of Medical Sciences and Peking Union Medical College.

## Author contributions

**Conceptualization:** Shuzhong Liu, Xi Zhou, An Song, Yipeng Wang, Yong Liu.

**Funding acquisition:** Shuzhong Liu, Yipeng Wang, Yong Liu.

**Investigation:** Shuzhong Liu, Xi Zhou, An Song, Zhen Huo, Yong Liu.

**Resources:** Shuzhong Liu, Xi Zhou, Zhen Huo, Yipeng Wang, Yong Liu.

**Supervision:** Yipeng Wang, Yong Liu.

**Writing – original draft:** Shuzhong Liu, Xi Zhou, An Song, Yong Liu.

**Writing – review & editing:** Shuzhong Liu, An Song, Yipeng Wang, Yong Liu.
